# Mucin Protein of Aedes aegypti Interacts with Dengue Virus 2 and Influences Viral Infection

**DOI:** 10.1128/spectrum.02503-22

**Published:** 2023-02-27

**Authors:** Karuna Yadav, Vipin Singh Rana, Anjali, Gunjan Kumar Saurav, Nitish Rawat, Ankit Kumar, Sujatha Sunil, Om P. Singh, Raman Rajagopal

**Affiliations:** a Gut Biology Laboratory, Department of Zoology, University of Delhi, New Delhi, India; b Department of Veterinary Medicine, University of Maryland, College Park, Maryland, USA; c Department of Zoology, Rajiv Gandhi University, Doimukh, Arunachal Pradesh, India; d Vector Borne Disease Group, International Centre for Genetic Engineering and Biotechnology, New Delhi, India; e National Institute of Malaria Research, New Delhi, India; Indian Institute of Science Bangalore

**Keywords:** *Aedes aegypti*, dengue virus, Interacting protein partners

## Abstract

Dengue, caused by dengue virus (DENV), is the most prevalent vector-borne viral disease, posing a serious health concern to 2.5 billion people worldwide. DENV is primarily transmitted among humans by its mosquito vector Aedes aegypti; hence, the identification of a novel dengue virus receptor in mosquitoes is critical for the development of new anti-mosquito measures. In the current study, we have identified peptides which potentially interact with the surface of the virion particles and facilitate virus infection and movement during their life cycle in the mosquito vector. To identify these candidate proteins, we performed phage-display library screening against domain III of the envelope protein (EDIII), which plays an essential role during host cell receptor binding for viral entry. The mucin protein, which shared sequence similarity with the peptide identified in the screening, was cloned, expressed, and purified for *in vitro* interaction studies. Using *in vitro* pulldown and virus overlay protein-binding assay (VOPBA), we confirmed the positive interaction of mucin with purified EDIII and whole virion particles. Finally, blocking of mucin protein with anti-mucin antibodies partially reduced DENV titers in infected mosquitos. Moreover, mucin protein was found to be localized in the midgut of *Ae. aegypti*.

**IMPORTANCE** Identification of interacting protein partners of DENV in the insect vector Aedes aegypti is crucial for designing vector control-based strategies and for understanding the molecular mechanism DENV uses to modulate the host, gain entry, and survive successfully. Similar proteins can be used in generating transmission-blocking vaccines.

## INTRODUCTION

Dengue virus (DENV) is an arbovirus (arthropod-borne virus) primarily transmitted by the insect vector Aedes aegypti (1). The symptoms of DENV infection range from mild fever to severe clinical manifestations, including dengue hemorrhagic fever and dengue shock syndrome (2). Its insect vector has a wide geographic range, exposing one-third of the world’s population to any of the four serotypes of DENV (DENV 1 to 4). Nearly 400 million people, mainly those living in tropical and subtropical countries, are at risk for DENV infection (3). Infection with one serotype gives lifelong immunity to the same serotype but induces susceptibility to the other serotypes, producing cross-reactive antibodies in humans (4); thus reducing the possibility of finding effective common antiviral targets against all serotypes (5, 6).

The mosquito acquires DENV via direct feeding on an infected vertebrate host. The virus replicates inside the *Ae. aegypti* gut and migrates to the salivary gland via hemolymph (7). The concentration of virus in the blood meal has a direct and positive association with the proportion of vector midgut infections (8, 9). Similarly, viral titer is also one of the factors which determine whether the vector is infectious enough to transmit DENV (8).

DENV is a single-stranded, positive-polarity RNA virus nearly 11 kb in size. It encodes three structural (1) (envelope, capsid, membrane protein) and seven nonstructural proteins (NS1, NS2A, NS2B, NS3, NS4A, NS4B, NS5) (10). Furthermore, the DENV genome is flanked by two untranslated regions (5′ and 3′ UTRs) which play important roles in viral transmission, replication, and modulation of the immune system (11).

DENV infects the host by binding to cell-surface receptors on the target cells and is internalized by receptor-mediated endocytosis. The envelope protein (E) plays a major role in receptor binding. It interacts with various cell surface receptors to initiate virus acquisition by the host cell. Crystallographic studies of E protein have shown the presence of three structurally different domains, named EDI, EDII, and EDIII (12, 13). EDI acts as the central domain, has a hinge region, and is responsible for structural changes in E protein in response to changing external pH. EDII has a hydrophobic region, which plays a key role in membrane fusion and dimerization of E protein, and thus acts as a dimerization domain. EDIII is thought to play a significant role in binding to the cognate receptor on the host cell surface (14–16). A number of DENV receptors have been proposed in previous studies. For example, different carbohydrate molecules, such as sulfated glycosaminoglycans and glycosphingolipids, have been implicated as receptor or co-receptor molecules in mammalian cells, whereas the same proteins have been observed as potential candidate receptors in mosquito cells (17, 18). The virus-host interactome involves complex machinery in which many host proteins are exploited as receptors and co-receptors. Hence, the identification and characterization of these proteins is imperative to increase knowledge concerning these interactions, which can be employed to devise a conceivable alternate therapy (19).

Dengue antiviral therapy and traditional vaccines have produced underwhelming results as treatment strategies (20). One possible approach that needs reconnoitering is the development of transmission-blocking vaccines, which requires identification of the interacting vector proteins (21). EDIII is an immunoglobulin (Ig)-like domain present at the C terminus. The EDIII domain is exposed on the virion surface and hence is accessible during attachment to the host cell receptor. It plays a role in recognition of the receptors or epitopes present on host cells. Studies of the structure-function relationship of DENV E protein domains using monoclonal antibodies against the EDIII domain have shown that EDIII is the most potent blocker of DENV infection (22). A similar study of domains I/II resulted in weakly neutralizing and cross-reactive antibodies against various flaviviruses that eventually enhance the chances of developing antibody-dependent enhancement (ADE) (23). Of all three domains, EDIII has antigenic properties and is mainly responsible for inducing long-lasting immunity against DENV infection. In conclusion, the EDIII domain has a crucial role in the disease’s pathogenesis and immunity. Thus, EDIII can act as most important protein to identify potential interacting peptides against DENV (12, 15, 16). Along this line, we focused on identifying and characterizing possible interacting vector-derived protein partners of the envelope protein domain III (EDIII) of DENV-2. To achieve this, we performed phage display library screening, and interacting peptide sequences of the bacteriophages were searched against the mosquito protein database on NCBI. Sequence analysis of positive clones led to the identification of putative candidates and their similarity to different protein families. The mucin-like protein was selected to further investigate its role as an interacting partner of DENV. The *mucin* gene was cloned into an Escherichia coli (E. coli) based-heterologous expression system and the protein was subsequently purified. The interaction between the mucin protein and the DENV was determined and validated using both *in vitro* and *in vivo* techniques. Furthermore, the role of mucin protein in DENV infection was evaluated by an antibody-blocking assay.

## RESULTS

### Phage display screening.

Phage display screening resulted in the identification of EDIII-interacting peptide sequences. These were subjected to the BLASTp 2.6.2 program in NCBI against *Ae. aegypti* protein collections. Several proteins were found as possible interacting partners/candidate receptors for EDIII of DENV-2 (Table 1). The titin and actin proteins, found in the phage display screening, are cytoskeletal proteins. Some proteins such as actin and endophilin B have been previously implicated in DENV-host interactions. Mucin proteins have been previously implicated in host-pathogen interactions and hence mucin protein was selected for further work.

**TABLE 1:** List of possible candidate receptors of dengue virus-2 (DENV-2) found in phage display screening

Protein	No. of hits	Accession no.	Start	Interacting peptide sequence
Titin	18	XP_001650724.1	935	STSFQS
Calmin	14	XP_001652989.1	146	PNKY
Cadherin	29	XP_001652662.1	84	LSKP
Toll	11	XP_001658496.1	191	SKGAG
Cathepsin B	04	XP_001658486.1	269	IWGH
Clathrin	05	XP_001660126.1	19	SNPFL
Endophilin B	02	XP_001660319.1	224	THLRH
Putative Salivary mucin	04	DQ439996.1	167	TEAPKPT
Serine protease	11	XP_001653721.1	465	SPYE
Actin	07	XP_001661194.1	43	PHSK

### Bioinformatic and phylogenetic analysis of *mucin*.

**FIG 1:**
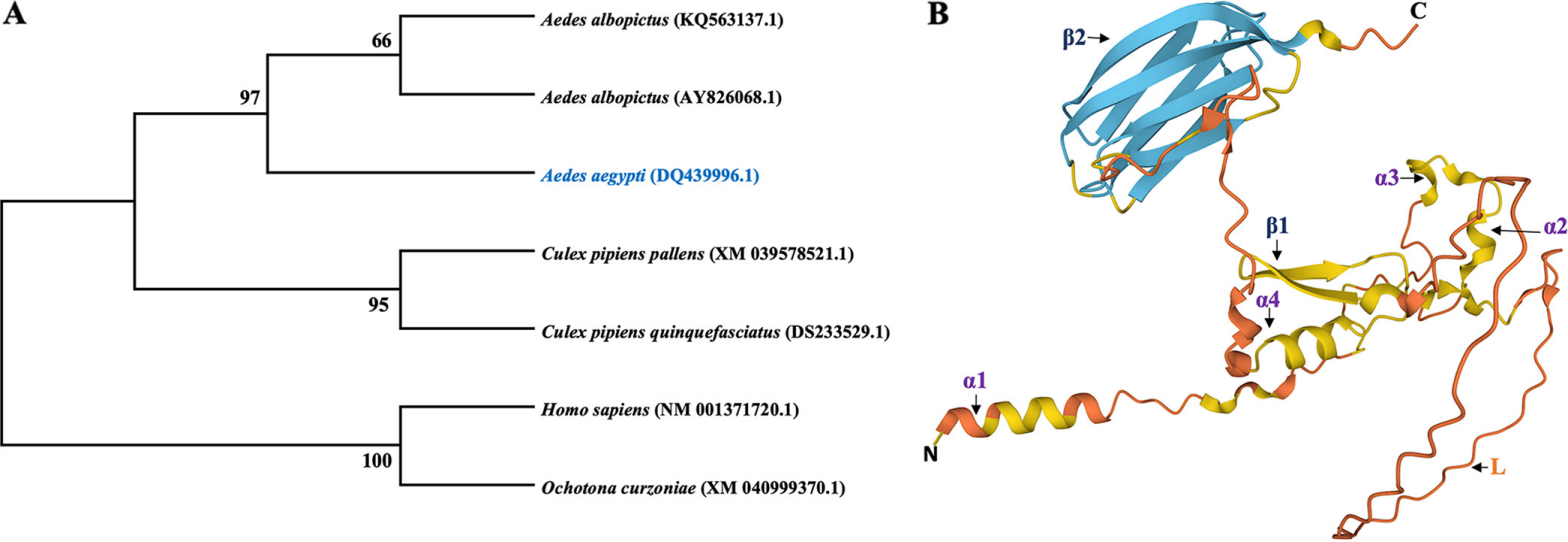
(A) Evolutionary analysis by Maximum Likelihood method. The evolutionary history was inferred using the Maximum Likelihood method and the Hasegawa-Kishino-Yano model. The tree with the highest log likelihood (−10,479.37) is shown. The percentage of trees in which the associated taxa clustered together is shown next to the branches. Initial tree(s) for the heuristic search were obtained automatically by applying Neighbor-Join and BioNJ algorithms to a matrix of pairwise distances estimated using the Maximum Composite Likelihood (MCL) approach and then selecting the topology with the superior log likelihood value. A discrete gamma distribution was used to model evolutionary rate differences among sites (5 categories [+G, parameter = 2.2239]). This analysis involved 7 nucleotide sequences. The final data set included a total of 3,702 positions. Evolutionary analyses were conducted in MEGA X. (B) Mucin protein structure prediction using the Alphafold protein structure database. Blue region indicates a per-residue confidence score (pLDDT) of >90, yellow region shows intermediate pLDDT (70 > pLDDT > 50), and orange region indicates pLDDT < 50. Secondary structures are assigned to the model (different alpha helix regions as α1, α2, α3, and α4; beta-sheet regions as β1 and β2; L represents the loop region).

Analysis of evolutionary relatedness among mucin sequences from mosquitoes and mammals (Homo sapiens, Ochotona curzoniae) showed that the *mucin* gene is also found in other mosquitoes (*Ae. albopictus*, Culex pipiens) and is closely related to the mucin of *Ae. aegypti.* However, this mucin was distantly related to the ones found in mammals (Fig. 1A). Analysis via InterProScan revealed a signal sequence from amino acids 1 to 19 and a non-cytoplasmic domain from amino acids 20 to 281, but no family membership was detected. SignalP also indicated presence of signal peptide in the mucin protein from amino acids 1 to 19 with a cleavage site between amino acids 19 and 20 (Fig. S1 in the supplemental material) (24). Secondary structural analysis of the mucin protein revealed 3% helix, 7% beta sheet, and 70% coil structure (Fig. S2). The mucin protein structure (AF-Q1HRU8-F1) was predicted using the Alphafold protein structure database as shown in Fig. 1B.

### Anti-mucin antibody was produced using purified recombinant mucin protein.

**FIG 2:**
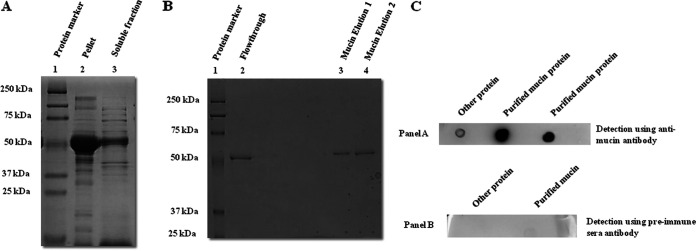
(A) SDS-PAGE analysis for purification of Trx-tagged mucin protein. Lane 1: protein marker (kDa). Lane 2: induced mucin protein in pellet fraction after sonication. Lane 3: induced mucin protein in soluble fraction after sonication. (B) SDS-PAGE analysis for elution of Trx-tagged mucin protein. Lane 1: protein marker (kDa). Lane 2: flowthrough after Ni-NTA agarose bead binding. Lanes 3 and 4: purified Trx-tagged mucin elution fractions (~50 kDa). (C) Dot blots of mucin protein with anti-mucin antibody produced in mice. Dot blot developed with antisera against mucin protein. Top lane: 5 μg other protein was blotted on nitrocellulose membrane along with 5 μg mucin protein (2 spots). Color was observed where mucin protein was blotted, whereas no color was observed with other protein. Bottom lane: dot blot developed with against pre-immune mucin. Here, 5 μg of other protein and mucin together was blotted on nitrocellulose membrane. No color was observed in the case of both proteins. This experiment indicated that antibodies against mucin were successfully generated as well as the specificity of generated antibodies.

The *mucin* gene was cloned into the pGEM-T Easy TA cloning vector. Restriction digestion was performed with NcoI and SalI restriction enzymes to confirm the cloning of *mucin*, which was later reconfirmed by commercial sequencing of the plasmid of the positive colony and its analysis using BLAST program of NCBI. The positive clone plasmid was further digested for the purpose of subcloning into pET-32a expression vector. E. coli BL-21 cells were transformed with the positive construct (pET-32a with *mucin* gene). The mucin protein along with Trx-tag was induced, expressed, and purified (Fig. 2A and B; Fig. S3). Various non-anionic detergents were used for the solubilization process, but these attempts failed. Finally, the cell pellet was treated with the detergent *N*-lauroylsarcosine. Similarly, MBP-EDIII protein and MBP-Tag alone were successfully expressed and purified in the bacterial expression system (Fig. S4). Anti-mucin antibody was produced in two BALB/c mice. Pre-immune sera from mice indicated no cross-reactivity against mucin protein in the dot-blot assay (Fig. 2C). Antisera were collected 7 days post-second and subsequent immunizations. Antisera tested positive against mucin protein in dot-blot assays (Fig. 2C). Antisera were aliquoted and stored at –20°C for further use.

### Mucin protein was expressed in *Ae. aegypti* midgut cells and co-localized with DENV-2.

**FIG 3:**
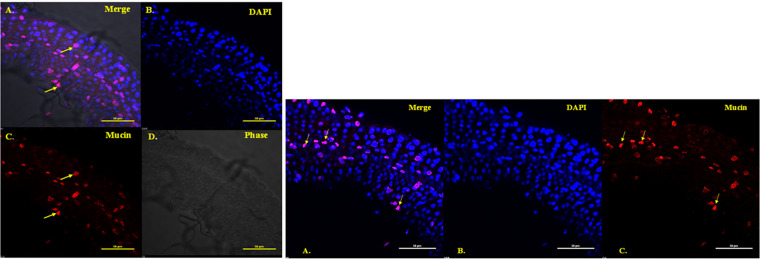
Confocal analysis for localization of mucin protein in midgut of Aedes aegypti. Panel 1 (left side): mucin protein expression in dissected midgut of *Ae. aegypti* at 40× magnification. (A) Merged image from panels B, C, and D. (B) Mucin expression was detected with primary anti-mucin antibody and secondary *Cy5*-conjugated anti-mouse antibody. (C) DAPI (4′,6-diamidino-2-phenylindole) (blue) staining for nucleus detection. (D) Bright field image. Panel 2 (right side): another view of *Aedes* midgut. (A) Merged image from panels B and C. (B) DAPI (blue) staining for nucleus detection. (C) Mucin expression was detected with primary anti-mucin antibody and secondary Cy5 conjugated anti-mouse antibody. Scale bar- 50 micron.

**FIG 4:**
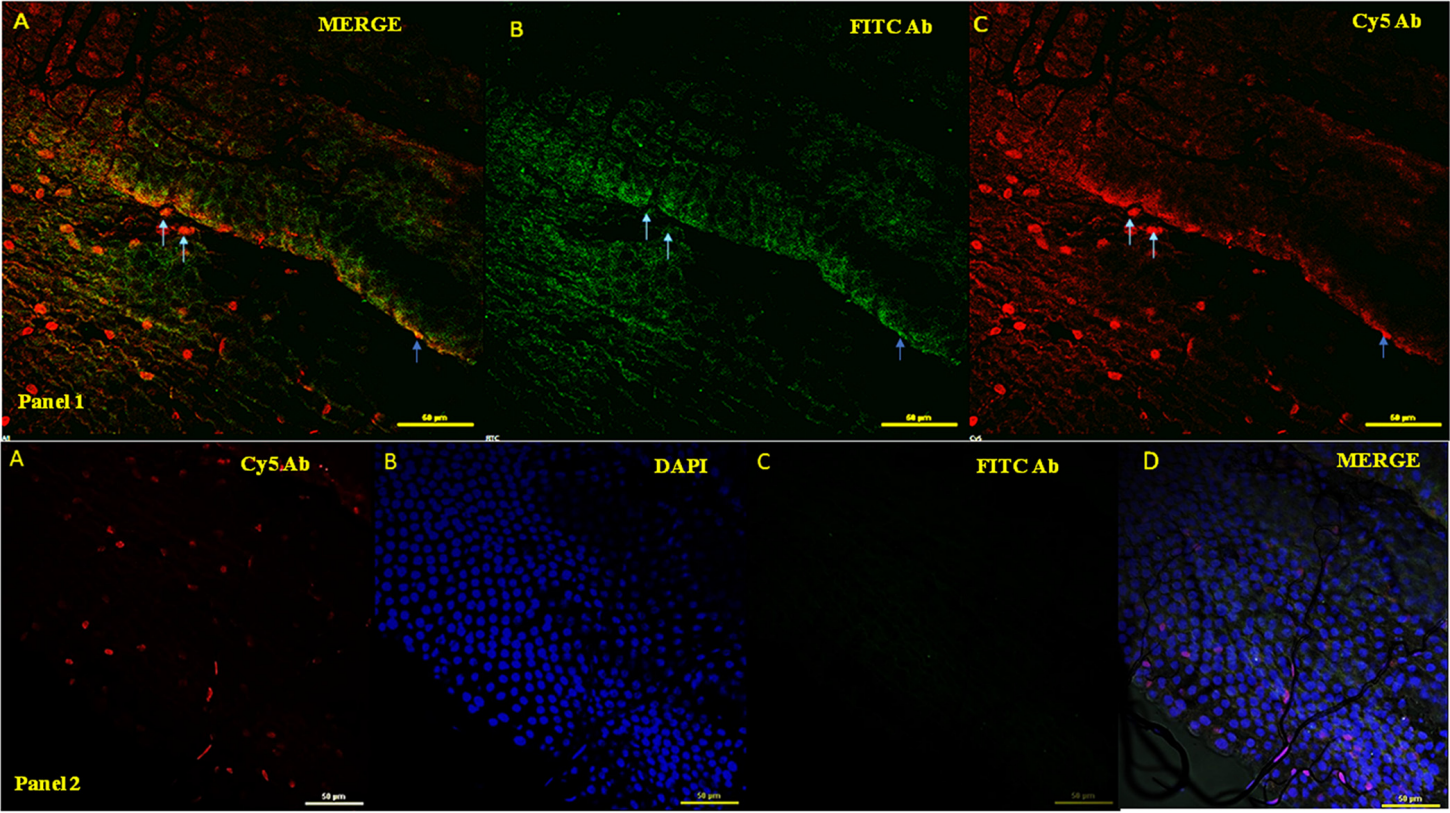
Confocal analysis for co-localization of mucin protein with dengue virus 2 (DENV-2) virion in gut of *Ae. aegypti*. Random gut locations shown in panel were analyzed for mucin protein expression. Panel 1 (top row): mucin protein expression in dissected gut of DENV-2 infected *Ae. aegypti* at 40× magnification. (A) Merged image from panels B and C. (B) Dengue virus detected with primary anti-dengue antibody (rabbit) and secondary FITC (fluorescein isothiocyanate)-conjugated anti-rabbit antibody. (C) Mucin protein expression (red) was detected with primary anti-mucin antibody and secondary *Cy5*-conjugated anti-mice antibody. Panel 2 (bottom row): as control experiment, healthy *Aedes* gut was incubated with primary anti-mucin antibody and secondary *Cy5*-conjugated anti-mice antibody as well as secondary FITC-conjugated anti-rabbit antibody. (A) Mucin protein expression in dissected gut of healthy/uninfected *Ae. aegypti*. (B) DAPI (blue) staining for nucleus localization. (C) Midgut incubated with FITC-labeled anti-rabbit secondary antibody; no primary antibody was used. (D) Merged image from panels A, B, and C. Scale bar- 50 micron.

A localization assay was performed to check for the expression of *mucin*. Confocal imaging showed the presence of mucin protein within the midgut cells of the adult *Ae. aegypti* mosquito (Fig. 3). A co-localization assay was performed to check for *mucin* expression in DENV-2 infected *Ae. aegypti*. Confocal imaging showed the presence of mucin protein co-localized in midgut cells of the adult *Ae. aegypti* mosquito (Fig. 4). This co-localization indicates the probability that two proteins exist in the same or very nearby compartments, which further suggests the possibility of the two proteins interacting with each other.

### Mucin protein interacted with DENV particles isolated from infected C6/36.

**FIG 5:**
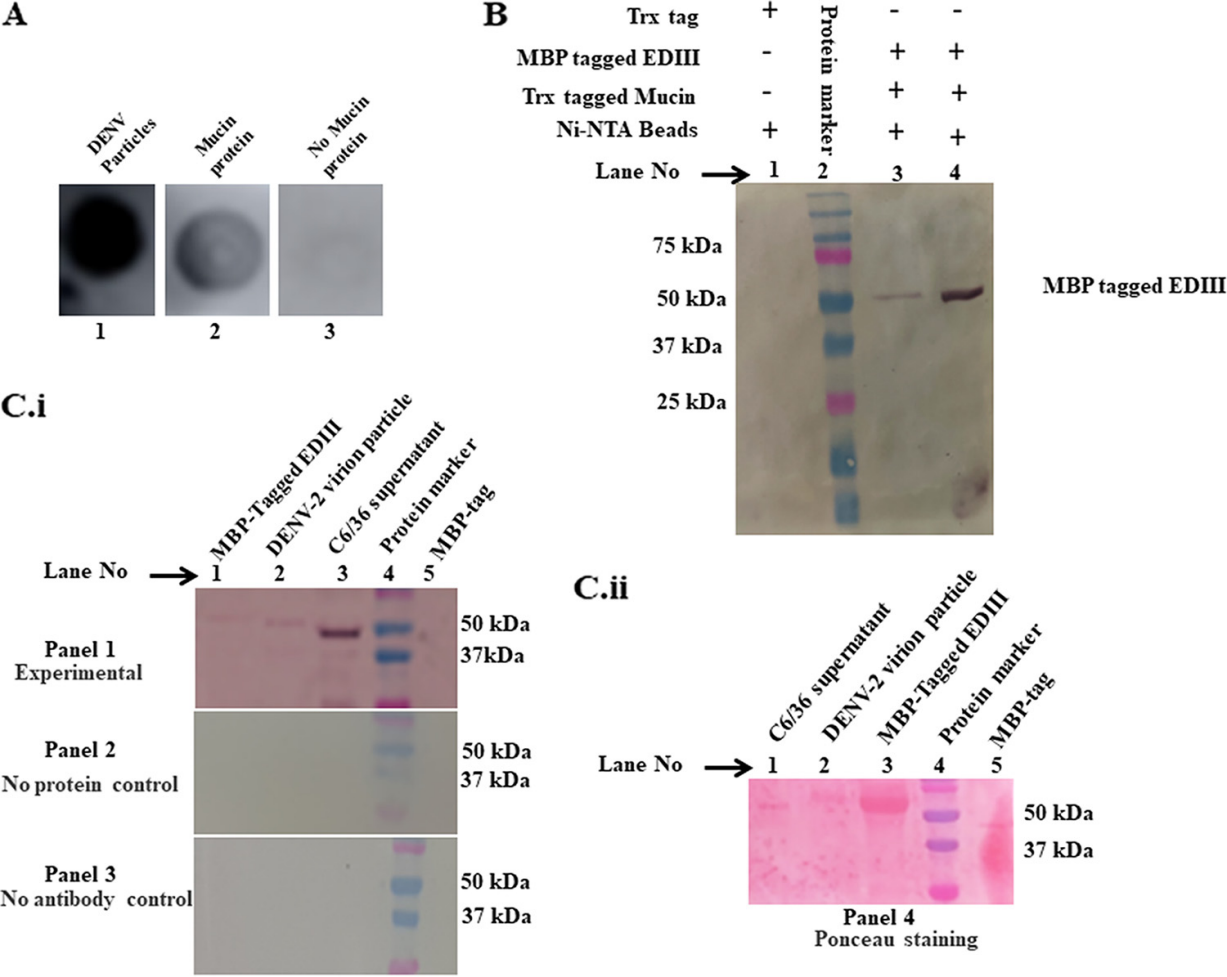
(A) Dot blot assay to check interaction of mucin protein with the DENV-2 particles. Image 1: positive-control mucin was not used for interaction with blotted DENV particles and blot was developed with anti-DENV antibody. Image 2: mucin protein was allowed to interact with the blotted DENV-2 particles. In both images 2 and 3, blots were developed with anti-mucin antibody Image 3: negative-control; no mucin was used to interact with the blotted DENV-2 particles. (B) Western blot of *in vitro* pulldown assay check interaction of His-tagged mucin with MBP-tagged EDIII. Lane 1: control-bound fraction of proteins. Lane 2: protein marker (kDa). Lane 3: Unbound fraction of proteins. Lane 4: bound fraction of proteins. In lanes 3 and 4, MBP-EDIII protein (53 kDa) was detected using anti-dengue antibody. (C) Virus overlay protein-binding assay (VOPBA). (i) Nitrocellulose membrane was blotted with MBP tagged EDIII, DENV-2 particles, C6/36 supernatant and MBP tag were incubated as in lanes 1 and 2 and lanes 3 and 5, respectively. Blotted nitrocellulosemembrane was incubated with purified mucin protein. Mucin protein was detected using primary anti-mucin antibodies and secondary anti-mouse ALP labeled antibodies. Experimental Lane 1: a band of 53 kDa was observed, suggesting positive interaction of mucin protein to EDIII. Lane 2: a band of ~54 kDa was observed suggesting direct interaction of mucin with virion particles. Lane 3: a band of ~52 kDa was observed, suggesting mucin protein directly interacts with envelope protein (E) of DENV-2. Lane 4: no band was observed, suggesting that MBP tag alone does not interact with mucin protein. (ii) Ponceau staining: lane 1, C6/36 supernatant; lane 2, DENV-2 particles; lane 3, MBP-tagged EDIII; lane 4, protein marker; lane 5. MBP tag.

To check the interaction of mucin protein with DENV-2 particles, a dot blot assay was performed (Fig. 5A). DENV-2 particles were isolated from the infected C6/36 cells supernatant and blotted on a nitrocellulose membrane. When the blotted DENV-2 particles were allowed to interact with mucin protein and anti-mucin antibody was used as the primary antibody, positive signals were observed. In contrast, in the negative control, where DENV-2 particles blotted on nitrocellulose membrane were not allowed to interact with mucin protein and blot was developed using anti-mucin antibodies, no signal was observed. In the positive control, the DENV-2-blotted membrane was not incubated with mucin and anti DENV antibody was used, resulting in a positive signal. These results clearly indicated that mucin interacts specifically with the DENV-2 particles isolated from C6/36 cells.

In a pulldown assay, the protein interaction with EDIII of DENV-2 was validated (Fig. 5B). The *in vitro* pulldown experiment results indicated the interaction of Trx-tagged mucin with maltose-binding protein (MBP)-tagged EDIII (53 kDa) of DENV-2. In the first control (only Trx-tag without mucin protein), no band was observed, indicating that Trx tag is not responsible for any interaction.

In the second approach to validate the interaction, a virus overlay protein-binding assay (VOPBA) was used to analyze the *in vitro* interaction of *Ae. aegypti* mucin protein with the envelope protein of DENV-2 (Fig. 5C, sections i and ii). The protein bands of ~53 kDa and ~54 kDa were observed with MBP-EDIII and virion particles, respectively. Bound mucin protein was detected using anti-mucin antibody. The results show an interaction between EDIII and mucin protein. No bands were observed with the MBP tag. In the case of the controls without primary anti-mucin antibody and those with no purified protein incubation, no significant bands were observed.

### Molecular docking validated the interaction of DENV with mucin protein.

**FIG 6:**
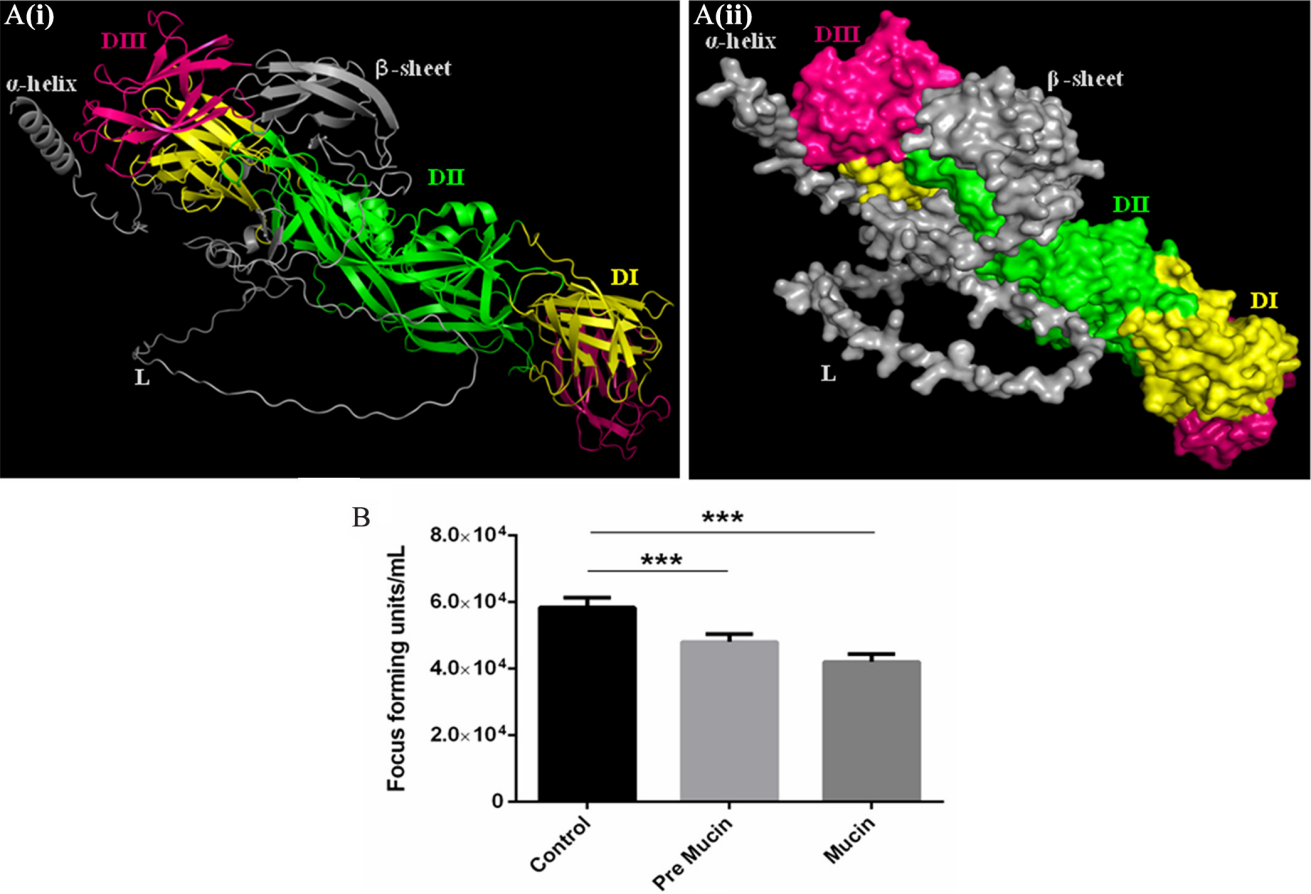
(A) Molecular docking for protein-protein interaction (i) Cartoon representation of protein-protein interaction between prey protein mucin (gray) and 1oa9 (envelope [E] protein) (EDI, yellow; EDII, green; EDIII, pink) (ii) Surface view of protein-protein interaction between prey protein mucin (gray) and 1oa9 (E protein). The prey protein mucin residues Asp22, Thr 34, Tyr 44, Tyr 45, Tyr 60, Glu 56, Ile 53, Gly 148, Ser 149, Tyr 50, Phe 21, Ile 155, and Pro 151 exhibit interaction with the residues Arg 286, His 282, Glu 269, Ala 267 Thr 265, Leu 264, Ala 259, Gln 256, Gly 223, Trp 220, Lys 204, Ile 170, Ile 129, and Arg 57 of 1OA9 protein. (B) DENV focus-forming assay. To assess the role of mucin protein in DENV-2 infection in *Ae. aegypti*, DENV-2 particles were microinjected into mosquitoes in three combinations (DENV + phosphate-buffered saline [PBS], DENV + pre-immune serum, and DENV + anti-mucin antibodies). At 6 days after microinjections, DENV-2 titers were quantified using focus-forming assay. Mosquitoes injected with DENV-2 and anti-mucin antibodies showed nearly 12.50% reduction in titers compared to control groups (DENV-2 + PBS or DENV-2 + Pre-immune serum). Statistical significance was checked using one-way analysis of variance (ANOVA) and unpaired *t* test (***, *P* < 0.001). This experiment was performed three times independently; each time, at least 5 individual mosquito whole bodies were evaluated for DENV-2 titers for each group.

The structure of the mucin protein was predicted by using Alphafold protein structure database. The docking experiments results were evaluated and the pose with least pose energy was selected. Furthermore, this mucin structure was subjected to the Schrodinger Maestro platform to dock with DENV envelope protein. Pose 1 exhibited the smallest pose energy of −1,049.037 and a pose score of −461.146. Since non-covalent bonds are essential to form the complex, the interaction distance between the two proteins was observed and interacting amino acid residues with lengths closest to the ideal bond length were considered. The interacting residues of prey protein mucin accounts to Asp22, Thr 34, Tyr 44, Tyr 45, Tyr 60, Glu 56, Ile 53, Gly 148, Ser 149, Tyr 50, Phe 21, Ile 155, and Pro 151 with the residues Arg 286, His 282, Glu 269, Ala 267, Thr 265, Leu 264, Ala 259, Gln 256, Gly 223, Trp 220, Lys 204, Ile 170, Ile 129, and Arg 57 of envelope protein of DENV (PBD:1OA9) (Fig. 6A). This docking result significantly validated the interaction of DENV with the mucin protein of *Ae. aegypti*.

Furthermore, to evaluate whether this binding was able to block DENV infection in the mosquitoes, DENV-2 particles were injected into *Ae. aegypti* along with anti-mucin antibody. In the control experiments, pre-immune sera and phosphate-buffered saline (PBS) were injected along with DENV-2 particles in a separate experiment. After 6 days of microinjection, a focus-forming assay (FFA) was performed to quantify the DENV-2 titers. The FFA revealed that *Ae. aegypti* injected with DENV-2 particles along with anti-mucin antibodies showed partial reduction of the virus, a 12.50% (one way analysis of variance [ANOVA], *P* < 0.001) reduction in DENV-2 titer, compared with control experiments (Fig. 6B).

## DISCUSSION

DENV causes one of the most important vector-borne diseases in humans, and it is primarily transmitted by *Ae. aegypti* mosquito. *Ae. aegypti* is highly anthropogenic and prevalent in tropical and subtropical regions (8). The geographical areas of DENV transmission have expanded in recent years and there has been a drastic increase in the incidence of dengue infections across the globe. The prevalence of all four serotypes of DENV has observed across all continents, including the Americas, Africa, and Asia (5). Despite endless efforts, there are very few methods to check the spread of the disease, which necessitates understanding of the molecular mechanisms of DENV replication in the insect vector. This molecular understanding with respect to viral replication and transmission inside vector will at least provide insights into theoretically possible strategies which target interacting vector proteins and different cellular pathways of vectors (17, 19). Upon feeding on DENV-infected human blood, *Ae. aegypti* ingests DENV particles into its alimentary canal along with blood. DENV particles subsequently reach the midgut region, overcoming the midgut infection barrier to establish infection. DENV first replicates in the midgut epithelium and then escapes the midgut to infect other tissues, further replicating in these tissues to establish infection there. Finally, DENV settles in the salivary glands before being ejected into the circulatory system of a host along with saliva during the blood meal to begin the next infection cycle (7, 25). Therefore, for devising a vector-based mitigation strategy, the midgut and salivary gland are the best sites for inhibiting the DENV life cycle in the insect vector (26). Hence, the identification and characterization of a possible candidate receptor and/or co-receptor proteins or protein-interacting partners of DENV from the midgut and salivary gland regions are crucial for developing a vector-based control strategy.

To identify these proteins of *Ae. aegypti*, we performed phage-display screening against EDIII of DENV-2. Phage display is a very important biotechnology tool that helps in analysis of protein-protein and protein-ligand interactions, improving the affinity of a protein to its receptor, constructing antibody fragments and antibodies, developing novel vaccines, and identifying membrane receptors of cancer cells (27, 28). Phage display can provide new tools for defense against bacterial, fungal, and viral infection (28). For example, this technique helped identify peptides which bound to envelope glycoprotein and showed antiviral activities against DENV-2 (29). It was also used to select cry toxin variants against a new target insect, *Ae. aegypti* (30).

The various peptide sequences of EDIII-interacting bacteriophages were found in a phage display assay and were searched for similarity against the mosquito protein database on NCBI. Several possible candidate receptors/protein interacting partners were found, one of which was mucin (Table 1). Mucin proteins are group of glycoproteins classified as secretory or membrane-associated proteins and are abundantly expressed in the digestive and respiratory tracts (31). SignalP analysis indicated the presence of signal peptide in the mucin protein from amino acids 1 to 19, which is characteristic of membrane or secretory proteins (Fig. S1). Mucin plays a variety of important functions, such as protecting tissue against biological and physical damage (32), regulating cell attachment (33), suppressing immune cell functions (33), and protecting cells and protein from proteolysis (34). These functions of mucin protein made it a suitable candidate for further investigation. The *mucin* gene was cloned and heterologously expressed in an E. coli expression system (Fig. 2A and B, Fig. S3). The purified protein fraction was used to generate polyclonal antibodies in mice (Fig. 2C). These antibodies were used for the detection and localization of the mucin protein in *Ae. aegypti*. The results of localization by confocal microscopy indicated the presence of mucin in the midgut (Fig. 3). A co-localization study indicated that mucin protein was expressed in the vicinity of DENV-2 virions, suggesting a possible interaction between these two (Fig. 4). As suggested from previous studies, mucin and mucin-like proteins are highly glycosylated, modified post-translationally, and *N*-acetyl galactosamine residues are present in the mature form of these protein (35). Because the E. coli expression system lacks the ability to naturally produce *N*- or *O*- glycosylation sites (36), the produced protein must be missing some key carbohydrate residues which play an indispensable role in this interaction (35). Therefore, the EDIII-mucin interaction was verified using a dot-blot assay and VOPBA, both of which showed positive results (Fig. 5A and B, Fig. 5C panels i and ii). VOPBA has been previously used to verify the interaction of DENV-2 with the possible candidate receptor proteins prohibitin, R80, and R67 in *Ae. aegypti* midgut cells and C6/36 cells (37–39). *In silico* molecular modeling of mucin protein-E protein interaction suggests that the interacting residues of prey protein mucin accounts to the Asp22, Thr 34, Tyr 44, Tyr 45, Tyr 60, Glu 56, Ile 53, Gly 148, Ser 149, Tyr 50, Phe 21, Ile 155, and Pro 151 with the residues Arg 286, His 282, Glu 269, Ala 267, Thr 265, Leu 264, Ala 259, Gln 256, Gly 223, Trp 220, Lys 204, Ile 170, Ile 129, and Arg 57 of 1OA9 (Fig. 6A). This docking result significantly validated the interaction of DENV with the mucin protein of *Ae. aegypti* in dot-blot assay and VOPBA. The impact of polyclonal antibody-mediated blocking of mucin protein on DENV titers in *Aedes* mosquitoes was statistically significant, although the reduction in DENV titer was minor. Previous studies have reported partial or even minor inhibitions of viral titers and have postulated that these inhibitions are potentially important for understanding the establishment and progression of infections in any model system (40–43). Because mucin is a glycoprotein and has signal peptide at the N terminus, DENV binding to mucin protein could be important for attachment and subsequent internalization of DENV into the midgut epithelium, where it replicates further before surpassing the midgut barrier.

Different mucin like proteins are predicted in vertebrates and invertebrates. There was a significant increase in mucin protein expression in the midgut, ovary, and fat body in *Ae. albopictus* after blood feeding. It has been suggested that mucin plays an important role in digestion, intestinal defense against gut-invading pathogens, and reproduction upon blood feeding (44). In *Drosophila*, mucin proteins are reported to be expressed during embryonic development. In a number of studies, mucin-like proteins were found to be involved in the formation of salivary sheath in various insects (Nilaparvata lugens, Laodelphax striatellus, and Nephotettix cincticeps) (45–48). This unique expression has been observed in the salivary glands of N. lugens (48) and Sogatella furcifera (49, 50), indicating their roles in feeding, oviposition, and interaction with the host plant (51). In the case of Anopheles gambiae, AgMuc1 protein has been reported to play important roles in protecting the intestinal epithelium and in interacting with the malarial parasite during midgut invasion (mosquito-parasite interaction) (52, 53). Nevertheless, the roles of different mucin proteins are still not clear in invertebrates. Different salivary mucins, Ctar-261 and Ctar-581, have been reported in Culex quinquefasciatus and *Aedes*; their expression is induced upon viral infection, suggesting the possibility of an immune function (35).

Understanding the interaction of DENV with its vector *Ae. aegypti* and identification of different potential receptors or interacting protein partners will open up various aspects to control DENV transmission by *Ae. aegypti.* Although the reduction in DENV titers is only partial upon blocking of mucin protein using our strategy, this study indicates that blocking the activities of mucin and mucin-like proteins may give us an opportunity to prevent the spread of the virus in human populations. However, specific functional analysis of these proteins is needed to develop novel vector control and management strategies in future.

## MATERIALS AND METHODS

### Mosquito rearing and collection.

*Ae. aegypti* (Delhi strain) were collected from the mosquito culture facility at the National Institute of Malaria Research (NIMR), Delhi, India. *Ae. aegypti* populations were reared at 27 ± 1°C with 70% to 80% relative humidity according to standard *Aedes* rearing protocol. Collected *Ae. aegypti* samples were stored in RNAlater (Ambion, Austin, TX) and kept at –70°C for subsequent use.

### Phage display library screening against EDIII.

To perform the phage display screening, a 150-μL volume of 100 μg/mL His-tagged EDIII protein (ProSpec) solution was coated on a single well of an enzyme-linked immunosorbent assay (ELISA) plate and used as the target against the commercially available Ph.D.-C7C Phage Display Library (New England Biolabs [NEB], Ipswich, MA), which contains a library of bacteriophages that express a unique 7-amino-acid-long random sequence peptide on their surface. The entire procedure was performed per the manufacturer’s instructions. In short, the phage library was incubated with purified protein EDIII protein overnight at 4°C. Following incubation, unbound phages were removed with stringent washes, whereas bound phages were eluted by lowering the pH. Eluted phages were re-amplified through an additional binding and amplification cycle (panning) to enrich the pool of strongly binding phages. In total, three rounds of panning were performed. To rule out the possibility of nonspecific binding, the ELISA well was washed six times with stringent wash buffer (Tris-buffered saline [TBS] containing 0.5% Tween 20 and 150 mM NaCl). Using a plaque assay, DNA was isolated from individual plaque and commercially sequenced (Macrogen, South Korea). The resulting nucleotide sequences were first translated to peptides using serial cloner and then searched for similarity against the *Ae. aegypti* protein database available in the NCBI server using the BLASTp program. Mosquito proteins showing similarity to peptides were selected for future work. The genes of the respective *Ae. aegypti* proteins were amplified, cloned, and heterologously expressed using a bacterial expression system.

### Bioinformatics.

The *mucin* sequences from the NCBI database were obtained for phylogenetic analysis. The unrooted phylogenetic tree was constructed with the Maximum Likelihood method by using MEGA X software with a bootstrap value of 1,000. The mucin sequence was subjected to InterProScan (https://www.ebi.ac.uk/interpro/search/sequence/) for functional module. The SignalP 4.1 server was used to detect the presence of signal peptide (http://www.cbs.dtu.dk/services/SignalP-4.1/). The amino acid sequence of mucin was subjected to Alphafold for structural prediction.

### Cloning, expression, and purification of mucin protein.

Total RNA was isolated from 10 female *Ae. aegypti* mosquitoes using Ribozol RNA extraction reagent (Amresco) as per the manufacturer’s instructions. It was used for cDNA synthesis with a RevertAid First Strand cDNA synthesis kit according to the manufacturer’s instructions (Thermo Fisher Scientific, Waltham, MA). Prepared cDNA was used for PCR amplification of *mucin* by using gene-specific forward primer, m2F (5′-GCCCATGGATGCAAACGTTTGCATGTATTTGTTTG-3′), and reverse primer, m2R (5′-CCGCTCGAGTCAACCAGAATTCCTGGTCGGGC-3′). The amplified PCR product of *mucin* of the desired size (846 bp) was eluted and cloned into pGEM-T Easy Vector (Promega, Madison, WI) by TA cloning and E. coli DH5α cells were transformed with these plasmids. Plasmid DNA was isolated from the positive colonies and sequenced. The *mucin* gene was subcloned into a pET-32a expression vector between the restriction sites NcoI and SalI, followed by transformation of the E. coli BL21 expression strain with the pET-32a-*mucin* construct. Transformed E. coli BL21 was grown in LB broth (containing ampicillin) at 37°C (180 rpm), until it reached an OD_600_ of 0.6 (optical density at 600 nm). Next, IPTG (isopropyl-β-D-thiogalactopyranoside) was added to a final concentration of 0.51 mM. It was incubated at 16°C (180 rpm) for 16 h to express the mucin protein. Next, 1-mL cultures of both induced and uninduced cells were harvested by centrifugation and suspended in 50 μL of SDS sample buffer. The samples were boiled for 5 min and 20 μL of each sample was loaded onto 12% SDS-PAGE gel. Gels were visualized using Coomassie brilliant blue (CBB).

### Cloning, expression, and purification of EDIII of dengue virus serotype 2.

The gene encoding EDIII of DENV-2 was synthesized commercially and cloned into a pMAL-c2X expression vector (New England Biolabs, Ipswich, MA) at the NdeI/SalI site. The E. coli BL-21 (DE3) expression strain was transformed with plasmid construct. EDIII protein was expressed by adding IPTG to LB culture at a final concentration of 0.01 mM at 16°C for 16 h. The induced culture cell pellet was re-suspended in lysis buffer (50 mM Tris-Cl [pH 7.5], 10% glycerol, 150 mM NaCl, 1 mM EDTA, 1× PI [GE Healthcare, Chicago, IL]) and lysed by sonication. The lysate was centrifuged at a high speed of 18,514 × *g* for 20 min at 4°C. The soluble fraction of lysate was incubated with pre-equilibrated Dextrin beads (GE Healthcare) in buffer for 2 h at 4°C on a nutator. After incubation, beads were washed twice with washing buffer [50 mM Tris-Cl (pH 7.5), 300 mM NaCl, 1 mM EDTA, 10% glycerol, 1× protease inhibitor, 1% Triton X-100 (vol/vol)]. Bound protein fractions were eluted with elution buffer (10 mM maltose [Sigma-Aldrich, St. Louis, MO], 50 mM Tris-Cl [pH 7.5], 150 mM NaCl, 1 mM EDTA, 10% glycerol, 1× protease inhibitor). Eluted fractions were checked on SDS-PAGE and dialyzed against native buffer. The dialyzed protein samples were stored at –70°C until further use.

### Anti-mucin antibody generation.

For anti-mucin antibody generation, two 6- to 8-week-old BALB/c mice were used. Pre-immune sera was collected from the retro-orbital area of each mouse a week before injecting the purified recombinant mucin protein as antigen. Mice were subcutaneously injected with 200 μg of purified recombinant-mucin protein. Mucin protein was emulsified in a 1:1 ratio with Freund’s complete adjuvant (Sigma-Aldrich, St. Louis, MO) for the first antigen injection, and Freund’s incomplete adjuvant (Sigma-Aldrich) was used for subsequent booster dosages. Blood was collected 7 days after injection of incomplete adjuvant booster and was kept at room temperature for 1 h and later stored at 4°C overnight to separate blood cells with the sera. The collected blood was centrifuged at 1,485 × *g* (Eppendorf 5810R) at 4°C for 10 min. Supernatant/antisera was collected in a microcentrifuge tube and aliquots were stored at –20°C. Collected antisera against mucin were checked with a dot-blot assay to determine reactivity against mucin protein.

### Localization of mucin protein with DENV.

For the localization of mucin protein in the insect vector, mosquito guts were dissected in 1× PBS under a stereomicroscope. Dissected guts were fixed in 4% paraformaldehyde (PFA) for 30 min and then permeabilized by incubating them with 1% PBST (1% PBS with 0.05% Triton X100) for an hour. Dissected guts were incubated with primary (1/200, anti-mucin produced in mice) and secondary (1/400 anti-mouse *Cy5* secondary antibody, Vectashield) antibodies for 2 h each, followed by blocking with 3% bovine serum albumin (BSA). Guts were washed thrice with 1% PBS after each incubation and were finally mounted on clean slides using Fluoroshield with DAPI (4′,6-diamidino-2-phenylindole; Sigma-Aldrich).

### Co-localization of mucin protein with DENV.

For co-localization, the mosquito guts were dissected in 1× PBS under a stereomicroscope. The guts were fixed in PFA for 30 min followed by permeabilization in Triton X-100 (1%) for 1 h. At each step, dissected guts were washed in 1× PBST. Guts were incubated with primary anti-mucin antibody (generated in mice) and anti-envelope antibody (generated in Rabbit, GTX127277) followed by incubation in secondary anti-mouse *Cy5* antibody and anti-rabbit FITC (fluorescein isothiocyanate) secondary antibody. At each antibody incubation, 3% BSA was used for blocking. After each incubation, guts were washed with 1× PBST. Guts were mounted on clean slides with DAPI Fluoroshield (Sigma-Aldrich). In the control experiment, healthy midgut was incubated with anti-mucin antibody (generated in mice), secondary anti-mouse *Cy5* antibody, and anti-rabbit FITC secondary antibody.

### Protein-protein interaction.

In the first approach, to analyze the interaction of mucin protein with purified DENV particles, a dot-blot assay was performed. DENV particles were purified from Aedes albopictus C6/36 DENV-2 infected cells. The purified particles were blotted on a nitrocellulose membrane. The membrane was blocked with 3% BSA for 1 h at room temperature (RT). Furthermore, the membrane was incubated with purified mucin protein for 1 h at RT. The membrane was washed thrice with 1% PBST (1% PBS + 0.05% Tween 20). The membrane was further incubated with primary anti-mucin antibody (1:3,000) for 1 h at RT. The membrane was washed twice with 1% PBST (1% PBS + 0.05% Tween 20). Horseradish peroxidase (HRP)-labeled secondary anti-mouse antibody (1:3,000 dilution) was used as secondary antibody and the membrane was incubated for 1 h at RT. Bands were visualized using an enhanced chemiluminescence system (Millipore, Burlington, MA) and documented through a gel documentation system (GE Healthcare). Negative and positive controls were also performed along with the experiment. As a positive control, DENV particles were not incubated with the purified mucin protein and detected using anti-dengue antibody. For the negative control, DENV particles were incubated with purified mucin protein and subsequently incubated with HRP-labeled secondary anti-mouse antibody (the blot was not incubated with primary anti-mucin antibody). The blots were visualized as described previously. In the second approach, a pulldown assay was performed. For this experiment, MBP-tagged EDIII purified protein was incubated with Trx-tagged mucin protein in binding buffer (10×) (25 mM Tris-Cl [pH 8], 5 mM MgCl_2_,75 mM NaCl, 2.5 mM DDT, 2.5 mM EDTA, and 1% NP-40). Two reactions were set, each reaction with equilibrated Ni-NTA beads. The reaction mixture was incubated at 37°C for 30 min. The reactions were centrifuged at 1,485 × *g* for 10 min at 4°C after a half-hour incubation. The unbound fraction was collected into a separate centrifuge. The beads were washed with washing buffer (binding buffer + 150 mM NaCl). Next, 30 μL of sample buffer was added to each reaction and the mixture was boiled at 100°C for 10 min. SDS-PAGE was performed and 25 μL of sample (after short spin) was loaded and analyzed on 12% gel. Gel was transferred to the nitrocellulose membrane and bands were detected using primary antibody (anti-dengue antibody, 1:3,000) for 1 h at RT. The blot was incubated for 2 h at RT in secondary antibody (Alkaline phosphatase (ALP)-labeled anti-mouse antibody, 1:2,000). The blot was developed using NBT-BCIP substrate. For further confirmation of the interaction between prey and bait protein, VOPBA was performed. For the VOPBA, DENV-infected C6/36 cell supernatant and purified MBP-tag, DENV virion particles, and MBP-EDIII were preheated with 2× SDS loading dye for 10 min in separate microcentrifuge tubes. After boiling, samples were run on 12% SDS-PAGE gel and further blotted on nitrocellulose membrane via Western blotting. The membrane was blocked using 3% BSA in 1% PBS for 1 h. The membrane was further probed/incubated with mucin protein for 2 h at 4°C. The membrane was washed twice with PBST. Afterwards, the membrane was incubated with the primary antibody, anti-mucin mouse antibody (1:3,000) for 1 h at RT. The blot was washed twice with 1% PBST. Furthermore, the membrane was incubated with ALP-labeled secondary anti-mouse antibody (1:3,000). The blot was developed using NBT-BCIP substrate.

### Molecular docking analysis.

The structure for mucin protein was obtained from Alphafold2, and the structure for dengue virus 2 E protein was retrieved from the Protein Data Bank (PDB). An FFT (Fast Fourier Transform)-based multi-stage PIPER tool from the Schrodinger Maestro platform (54) was used to analyze interacting amino acid contact points between targets A (mucin protein) and B (envelope protein of DENV), which confirms its interacting complex during molecular docking. The top model complex is expected to match the real-time scenario in which the PIPER code is supported by CAPRI (Critical Assessment of Prediction of Interactions) (55) blind experiments by evaluating the energy distribution of multiple docked conformations on a grid, ranking the model complex in a correlation function. All protein structures were loaded as PDB files in Schrödinger 2020 and prepared using the embedded Protein Preparation Wizard.

According to the PIPER method, the target protein is regarded a static macromolecule, and the remaining host proteins search for it every 5 degrees in the space of Euler angles using a 1.2-grid cell size (56, 57). A total of nine complexes are chosen for the analytical research, and top model complexes are computed for further analysis from each of the study sets. Each complex is examined carefully to evaluate the amino acid frequency distribution toward the binding site for assessment. The target protein’s higher-frequency residues show a potent active site map, which could be used to determine the possible interacting site.

### Blocking of mucin protein and DENV-2 infection in *Ae. aegypti*.

To block mucin protein and determine its effect on DENV transmission, we used 16 μg/μL of anti-mucin-antibody and 1.38 × 10^7^ focus-forming units (FFU) per mL of DENV-2 (ATCC VR-1584TM). The antibody and virus were mixed at a 1:1 ratio and 69 nL of this mixture was injected into the thorax of *Ae. aegypti* mosquitoes. In the control experiments, pre-immune sera and PBS were injected along with DENV-2. At 6th day post-injection, mosquitoes were analyzed for virus titer using a focus-forming assay (FFA). To conduct FFA, five mosquitoes were taken from each group and homogenized in 1 mL of Dulbecco’s modified Eagle medium (DMEM) separately. After centrifugation, supernatant was collected and filtered using 0.22-μm syringe filters. These samples were used to perform FFA for DENV-2 quantification using standard FFA protocols with slight modifications. Briefly, Vero cells were seeded in 96-well plates, followed by inoculation of the homogenized mosquito samples at different dilutions (starting at 1:50). Viral adsorption was allowed to proceed for 2 h at 37°C.

An overlay containing 5% fetal bovine serum (HiMedia RM10681) and 1% carboxymethyl cellulose (Sigma-Aldrich C4888) in DMEM was added at the end of adsorption. The infected monolayer was incubated at 37°C. After 72 h of infection, the overlay medium was removed from the wells, and cells were washed with cold PBS. The cells were fixed for 30 min in pre-chilled acetone: methanol (1:1). After washing with PBS, the cells were blocked with 5% skim milk (HiMedia GRM 1254)/PBS for 30 min. Infected cells were detected with a monoclonal anti-dengue antibody (Sigma-Aldrich SAB702232). After washing with PBS, antibody-labeled cells were detected with a secondary antibody conjugated to HRP (Novus Biologicals NB120-6808). The labeling was visualized with KPL TrueBlue peroxidase substrate (SeraCare 5510-0030). The FFUs were counted, and the viral titers were determined by times dilution factor. All experiments were performed at least three times. One-way ANOVA and an unpaired *t* test were performed using GraphPad Prism for statistical significance. Comparisons between control, pre-immune sera, and anti-mucin antibodies sera were made using one-way ANOVA (GraphPad 6, GraphPad software).
