# Supplementary material for: Mucin Protein of Aedes aegypti Interacts with Dengue Virus 2 and Influences Viral Infection
Source: Microbiol Spectr. 2023 Feb 27;11(2):e02503-22. doi: 10.1128/spectrum.02503-22 (PMC10101019; doi:10.1128/spectrum.02503-22)
Supplement: Supplemental file 1 — Fig. S1 to S4. Download spectrum.02503-22-s0001.pdf, PDF file, 1.0 MB [file spectrum.02503-22-s0001.pdf]

Title : Mucin protein of *Aedes aegypti* interacts to dengue virus-2 and influences viral infection

Authors: Karuna Yadav[a], Vipin Singh Rana[b], Anjali[a], Gunjan Kumar Saurav[c], Nitish Rawat[a], Ankit Kumar[d], Sujatha Sunil[d], Om P. Singh[e], Raman Rajagopal[a,*]

[a]- Gut Biology Laboratory, Room No. 117, Department of Zoology, University of Delhi, Delhi-110007, India

[b]- Department of Veterinary Medicine, University of Maryland, College Park, Maryland, USA

[c]- Department of Zoology, Rajiv Gandhi University, Doimukh, Arunachal Pradesh-791112, India

[d]- Vector Borne Disease Group, International Centre for Genetic Engineering and Biotechnology, New Delhi-110067, India

[e]- National Institute of Malaria Research, New Delhi-110077, India

Running Head: *Dengue virus-Aedes aegypti* interaction

[*]- Corresponding Author and Address:

Raman Rajagopal, Gut Biology Laboratory, Room No 117, Department of Zoology, University of Delhi, Delhi, India-110007.

E-mail: zoorajagopal@gmail.com

Telephone: 91-11- 27662275 / 27667985

Supplementary Materials

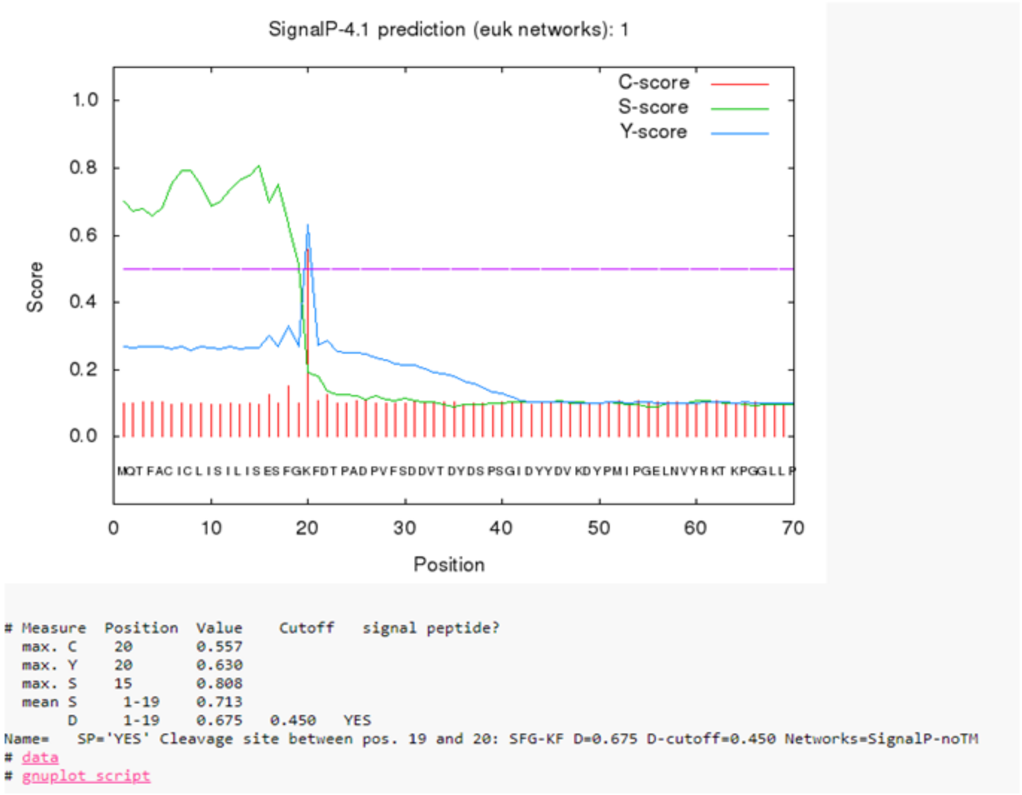

Fig S1: SignalP-4.1 for signal peptide property in mucin protein. Result indicated presence of signal peptide at 19 and 20 amino acid residues.

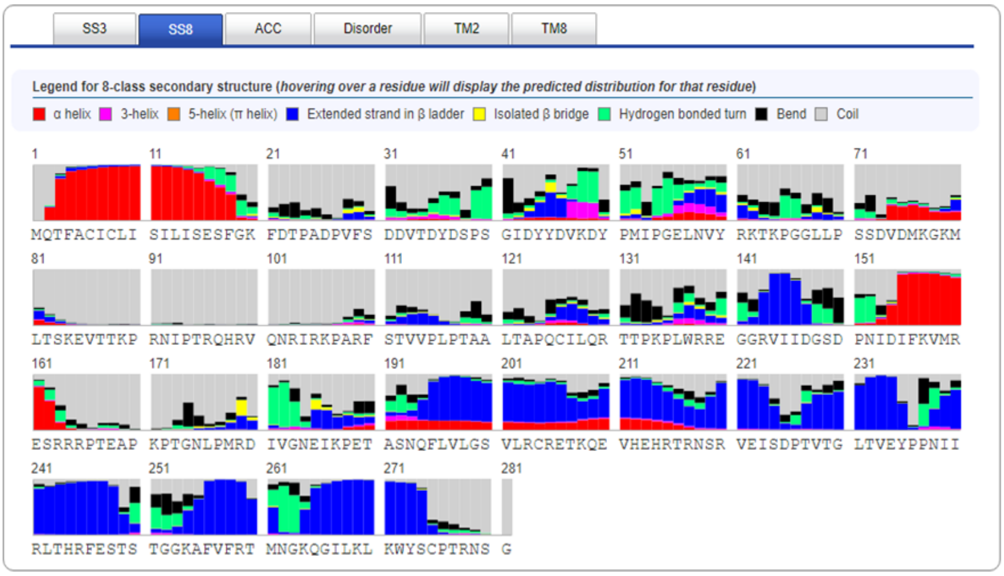

Fig S2: Eight-class secondary structure of mucin protein predicted by RaptorX. Different colors represent different secondary structure.

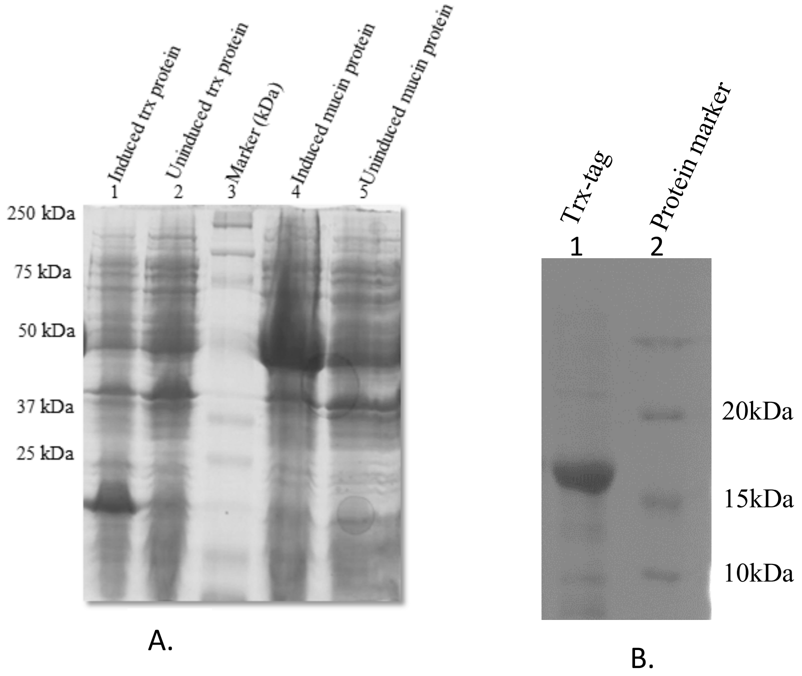

Fig S3: SDS-PAGE analysis of Trx-tagged mucin protein induction in E. coli BL21 [DE3] cells.A. Lane1- cell lysate of bacterial transformed with pET-32a vector 0.5 mM IPTG induction. **Lane 2**- cell lysate of bacterial transformed with pET-32a- vector without induction**. Lane 3**- Protein marker (kDa). **Lane 4**- cell lysate of bacterial transformed with pET-32a-mucin with 0.05 M IPTG induction. **Lane 5**- cell lysate of bacterial transformed with pET-32a-mucin without induction. **B. Purification of Trx-tag.** SDS-PAGE analysis for elution of Trx-tag. **Lane 1**- Purified Trx tag (~17kDa). **Lane 2**- Protein marker

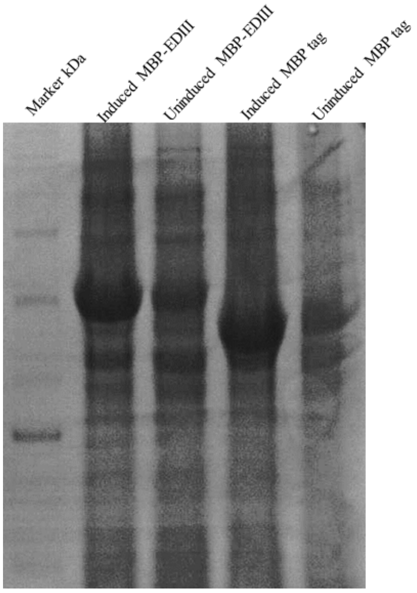

A

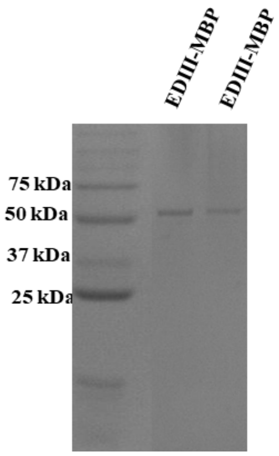

B

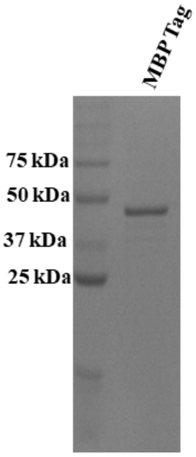

C.

Fig S4: A. **SDS-PAGE analysis of MBP-tagged EDIII Induced protein. Lane 1**- Protein marker (kDa). **Lane2**- cell lysate of bacterial transformed with pMAL-c2x-EDIII vector 0.01 mM IPTG induction. **Lane 3**- cell lysate of bacterial transformed with pMAL-c2x-EDIII vector without induction**. Lane 4**- cell lysate of bacterial transformed with pMAL-c2x with 0.01 mM IPTG induction. **Lane 5**- cell lysate of bacterial transformed with pMAL-c2x without induction. **B. Protein purification.** SDS-PAGE analysis for elution of MBP-EDIII protein. **Lane 1**- Protein marker (kDa). **Lane 2 & 3**- Purified MBP-EDIII protein. **C. Purified MBP Tag.** SDS-PAGE analysis for elution of MBP-tag protein. **Lane 1**- Protein marker (kDa). **Lane 2-** Purified MBP-tag.
